# Variations in Institutional Review Board Approval in the Implementation of an Improvement Research Study

**DOI:** 10.1155/2013/548591

**Published:** 2013-04-23

**Authors:** Darpan I. Patel, Kathleen R. Stevens, Frank Puga

**Affiliations:** Academic Center for Evidence-Based Practice, School of Nursing, University of Texas Health Science Center at San Antonio, MSC 7949, 7703 Floyd Curl Drive, San Antonio, TX 78229, USA

## Abstract

The purpose of this paper is to report the variance in institutional review board (IRB) reviews as part of the implementation of a multisite, quality improvement study through the Improvement Science Research Network (ISRN) and recommend strategies successful in procuring timely IRB approval. Using correspondence documents as data sources, the level of review was identified and time to submission, time to approval, and time to study start were analyzed. Thirteen of the 14 IRBs conducted independent reviews of the project. Twelve IRBs approved the study through expedited review while two IRBs reviewed the project at a full board meeting. Lastly, 11 of the 14 sites required documented consent. The greatest delay in approval was seen early on in the IRB process with site PIs averaging 45.1 ± 31.8 days to submit the study to the IRB. IRB approvals were relatively quick with an average of 14 ± 5.7 days to approval. The delay in study submission may be attributed to a lack of clear definitions and differing interpretations of the regulations that challenge researchers.

## 1. Introduction

With the push to increase the quantity, quality, and generalizability of improvement research [1–3], networks such as the Improvement Science Research Network (ISRN) provide an opportunity to conduct rigorous multisite studies; however, the inconsistency of review for improvement research brings challenges to both academic- and hospital- based IRBs. A formidable barrier to carrying out multisite improvement research is the IRB review process itself. Completion of the IRB application is a necessary yet time-consuming process [4]. The ISRN has developed a streamlined approach to facilitate IRB submissions at the local site through the use of the protocol implementation kit (PIK).

Quality improvement in the healthcare industry has gone through a major change. With the landmark report from the Institute of Medicine indicating the need to transform the healthcare system [2], quality improvement research must go beyond the single site, single investigator mindset. Using the implementation science framework focusing on participatory implementation process [5], many clinicians are working alongside their academic partners engaging in quality improvement activities to improve healthcare processes and patient outcomes. With the need to disseminate best practices, publication of quality improvement activities is warranted; however, many journals and publishers will not publish original data if the project was not approved by an accredited institutional review board. IRB review and federal agency oversight are increasing in importance as QI initiatives must rise to the level of research in order to facilitate dissemination and implementation of effective improvement strategies.

Title 45, part 46 of the Code of Federal Regulation (CFR) defines research as “a systematic investigation, including research development, testing and evaluation, designed to develop or contribute to generalizable knowledge [6].” Alternatively, quality improvement (QI) is defined as a systematic, data-guided activity designed to bring about immediate, positive changes in the delivery of healthcare in particular settings [7] or a process by which individuals work together to improve systems and processes with the intention to improve outcomes [8]. The Office for Human Research Protection (OHRP), the federal office governing IRBs, provides leadership in the protection of the rights, welfare, and wellbeing of participants involved in research conducted or supported by the US Department of Health and Human Services (DHHS) [9]. Therefore, as the governing office for IRBs, DHHS charges each IRB to assure, both in advance and by periodic review, that risk to human subjects is minimal and the welfare and rights of human subjects are protected [9].

Quality improvement may be successful within a single unit or hospital; however, the limiting factor with single site quality improvement projects is that it may not yield generalizable knowledge that can be implemented into clinical settings [1, 3]. Therefore, to address the issue of generalizability, the Improvement Science Research Network (ISRN) was created [9, 10]. The ISRN was created through the National Institutes of Health funding in 2009 as a national research infrastructure to advance improvement research. The ISRN is made up of nearly 200 academicians and clinicians from across the country, a cyberinfrastructure supporting virtual collaboration, and a research coordinating center. The mission of the network is to advance the scientific foundation for quality improvement, safety, and efficiency through transdisciplinary research addressing healthcare systems, patient-centeredness, and integration of evidence into practice [7]. In addressing its mission, the ISRN's infrastructure is tailored to conduct multisite improvement research to produce generalizable knowledge. 

 Previous publications have documented variations of IRB review for research networks engaged in multisite clinical trials [4, 11–15]. A large, high profile study to reduce central line-associated bloodstream infection (CLABSI) in the state of Michigan may have initiated the debate of whether a project is a QI initiative or a research study involving human subjects [16]. This study aimed to improve culture within the hospital and implement evidence-based practice to prevent CLABSI. This study was reviewed by the John Hopkins IRB and was considered exempted from further review. After the study report was published, OHRP indicated that Hopkins and the participating hospitals should have obtained full IRB approval with patient consent prior to initiating this study. This action by OHRP greatly changed the way quality improvement is viewed. The purpose of this report is to provide an ad hoc, descriptive review of the process required to obtain IRB approvals for the implementation of a multisite, quality improvement study.

### 1.1. Objectives of the Multisite Study

Small troubles, adaptive responses (STAR-2): frontline nurse engagement in quality improvement (PIs: Kathleen R. Stevens, RN, EdD and Robert Ferrer, MD, MPH) was a multisite, cross-sectional, multivariate research study aimed to describe the type and frequency of operational failures (or workarounds) detected by frontline nurses on their clinical units [17]. To complete this project, ISRN PIs partnered with 14 hospitals from across the country. Each site, led by a site PI, engaged nurses from three medical-surgical units to self-report operational failures encountered in routine care, in real time, using an index sized “Pocket Card” for 10 shifts over 20 days. Subsequently, frontline engagement, work environment, and quality improvement outcomes data were collected using an integrally designed survey packet. In total, 716 nurses participated from the 41 units involved in this study. This study start was staggered and broken into three waves. Wave 1 consisted of 2 hospitals, while waves 2 and 3 consisted of 6 hospitals each.

## 2. Methods

### 2.1. Protocol Fidelity

To assure that scientific rigor and protocol fidelity were maintained, the ISRN Coordinating Center, housed in the Academic Center for Evidence-Based Practice at UTHSCSA, coordinated this project. To facilitate fidelity of the project, a protocol implementation kit (PIK) specific to the STAR-2 study was provided to each site PI. The PIK included standardized materials to implement this project uniformly across each site. The PIK included the standardized protocol, marketing/advertising materials, IRB templates to facilitate the IRB submission process, step-by-step data collection processes, data entry guidelines, and tips to interpret results (see Puga et al.'s article on pages XX-XX for more detail) [18]. The UTHSCSA IRB approved this study prior to sending materials to each site.

### 2.2. IRB Approvals

**Figure 1:**
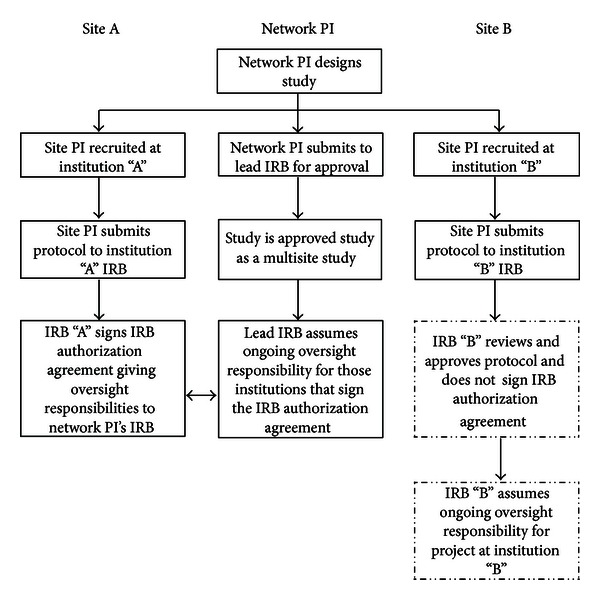
Overview of the multi-site research approval process.

This study was approved through expedited review by the University of Texas Health Science Center at San Antonio (UTHSCSA) Institutional Review Board (IRB) with verbal consent. Using materials from the initial approval as a template, the ISRN Coordinating Center developed a standardized protocol, IRB application, consent form, and other IRB materials for each site to facilitate IRB applications. These documents were sent along with the PIK to each site principal investigator for use in preparation of local IRB submissions. Each site was also requested to ask their local IRB to sign an investigator agreement to allow the UTHSCSA IRB and its Federalwide Assurance (FWA) to have oversight of the research site to speed up the implementation of the STAR-2 study. The ISRN Coordinating Center assisted each site PI in preparing for IRB submission and addressing any queries their IRB may have. If modifications to the protocol or consent form were required, standard operating procedures at the Coordinating Center stipulate that changes be implemented for the individual site only and not implemented at the other sites. Upon receiving local IRB approval, the site PI was asked to provide the ISRN Coordinating Center with the IRB approval letter prior to initiating study related procedures.  Figure 1 depicts the schematic for IRB approval process for the ISRN.

### 2.3. Data Collection on IRB Variation

Using the IRB application, correspondence, and approval letters as data, we identified the level of review conducted (exempt, expedited, or full board) and noted any changes that were made in the final approved documents relative to the standardized protocol and consent form. We calculated time to submission from the date the materials were received by the hospital to the date the IRB submission was made, time to approval from the date of IRB submission to IRB approval, and time to study start from the date IRB approval was recieved to the date the study began. 

### 2.4. Analysis

An ad hoc review of IRB application materials and correspondences was conducted. Where materials were unclear, the coordinating center contacted the site PI and/or local IRB for clarification. Statistical analysis is descriptive and presented as mean ± standard deviation.

## 3. Results

### 3.1. Type of Review

Each of the 14 sites received IRB approval from their IRB of record without revisions to the protocol, consent form, or resubmissions. All but one of the study sites conducted an independent review of the standardized protocol. The IRB of the study site that did not pursue independent review accepted the UTHSCSA IRB approval and signed an investigator agreement to fall under the UTHSCSA FWA. Of the fourteen hospitals that were engaged in this study, 4 used IRBs affiliated with local universities while the remaining 10 used IRBs located within their hospital or hospital system. 

 Twelve of the 14 (86%) study sites obtained IRB approval through an expedited process, with the remaining two study sites having the study go to full board review. Each of the expedited approvals were approved under 45 CRF 46.110(b)(1) category 7 [6]. Two of the sites that underwent expedited review had concerns about the database security. Standard procedures and accessibility guidelines were provided to these IRBs by the ISRN Coordinating Center and the study was ultimately approved. Twelve of the 14 IRBs required that consent be documented in the form of a consent form while 2 hospitals only required verbal consent.  Table 1 describes the type of IRB used, type of review conducted, and whether documented consent was required.

### 3.2. Approval Timelines

 The STAR-2 multisite study was implemented in three waves with two study sites in wave 1 and 6 study sites each in waves 2 and 3, respectively. Study materials were sent to all study sites on the same calendar date regardless of the study start date and study sites were asked to complete the IRB process as soon as possible to prevent delays in study startup.  Table 2 provides data on the approval timelines for time to submission, time to approval, and time to study start. On average, the 14 study sites submitted the project to their IRB 45.1 ± 31.8 days after receiving the study related documents, received approval 14.3 ± 5.7 days after submission, and started the study 29.9 ± 10.1 days after receiving IRB approval. There was no significant difference in approval times between academic-based IRBs and hospital-based IRBs.

## 4. Discussion

The purpose of this paper was to document variation in IRB review in the implementation of the STAR-2 multisite study conducted and coordinated by the ISRN. With 14 study site IRBs engaged in this study plus the UTHSCSA IRB of the ISRN Coordinating Center, there is a possibility that variations in review processes and time to approval would directly impact the study timeline. However, based on the results of this report, there were many similarities between IRBs with regard to the level of review and time to approval. We attribute this to the nature of the STAR-2 study and provision of the PIKs provided to each site PI to facilitate the submission of their IRB applications. Furthermore, support and interactions from the ISRN Coordinating Center along with the Network PIs (Stevens and Ferrer) assisted in guiding the site PIs in the IRB process and working with individual IRB process and addressing questions regarding IRB issues. Each PIK consisted of templates for IRB applications, consent forms, data collection tools, recruitment and marketing materials, and information to be presented to hospital executives to gain buy-in. These templates were based on the initial submission made by the ISRN Coordinating Center to the UTHSCSA IRB. By providing an IRB template, the site PIs and research associates simply had to modify certain components of the application specific to their IRB and were able to submit the IRB application as soon as 2 days after receiving the PIK.

The largest variance in the study timeline was seen between the three waves of the study. As indicated in Section 2, the STAR-2 study was implemented through three separate waves to allow for adequate oversight and assistance by the ISRN Coordinating Center. Two hospitals participated in the first wave, 6 in wave two, and the final 6 in wave three. Two of the hospitals (hospital H and hospital N) had to adjust their start-up time based on competing demands and arising priorities (e.g., local credentialing visits by Joint Commission and various other organizations). This accounted for the delays in their time to submission. However, once submitted, these two hospitals received IRB approval and started the study within one standard deviation of the average approval time. Eliminating these two sites as outliers, average time to submission was 33 days.

Looking at each of the academic IRBs individually, there was variation in the IRB's composition and structure. Hospital A's IRB of record chose to sign an investigator agreement falling under the regulatory oversight to the UTHSCSA IRB. Hospital B's IRB of record was a part of the faculty senate consisting of an IRB chair, 7 scientific reviewers, and one public reviewer. Hospital D and hospital M's IRB of record come from large universities with standing administrative review processes in place, including expedited/exempt review panels specific for each of the major schools in the program. Hospital M's IRB goes as far as having an online checklist to determine if the research projects require IRB approval. To facilitate a quick approval from each of the study sites at the onset of the STAR-2 study, each site was encouraged to sign investigator agreements to fall under the federalwide assurance of the parent IRB at the UTHSCSA. Though strongly encouraged, this agreement was done only by one hospital that participated in this study. The extremely low number of investigator agreements perhaps is due to oversight pressures felt by local IRBs to govern and regulate studies conducted at their respective institutions and an increase in regulatory actions [19]. In a multisite research study for the ISRN, the site PI is considered a partner on the investigative team and given the rights and responsibilities directed by DHHS. 

The level of review conducted and the time for this review by the local IRB were a concern for the ISRN Coordinating Center going into the implementation of this study. For example, if all 14 study site IRBs conducted full board reviews of the project, the study timeline for the entire project would be greatly affected and potentially delay study start up at these hospitals. More specifically, engaging employees of the hospital brings into account sensitivities in job performance and job security; however, because the focus of the study is centered on operational processes and system failures, review boards considered this minimal risk to the study participant as indicated by the number of expedited approvals. Additionally, the structure and process of documenting operational failures and system context were done so in an anonymous fashion, ensuring confidentiality for the protection of participants against risk and harm. 

An important consideration made by the review boards was the role supervisors would play in consenting staff they oversaw. The ISRN and the site PIs collaborated on ways to reduce undue influence by supervisors during the consent process. Working with the site PIs and their local IRB, a decision was made requiring supervisors not be involved in the consenting process. If consenting was to be done during a regular staff meeting, then the supervisor was asked to step out of the meeting. If the consenting was to be done individually, as time permitted, then research staff would be the ones consenting study participants. This was explicitly written into the protocol implementation kit as well as discussed in detail during the protocol training/study initiation meeting.

There are several limitations to this study. First, this is a descriptive study of 14 hospital IRBs evaluating one study and thus the results of this study may not be generalizable. Secondly, this report presents data based on a single QI study and may not represent how these IRBs would review other types of projects (i.e., drug trials involving patients, observational studies involving staff and patients, etc.). Though limitations to this study exist, this report presents important review of how 14 IRBs review a single study. Furthermore, the multisite study presented in this report was a minimal risk study of nurses self-reporting system operational failures with no interactions with patients. However, the operational failures have direct impact to patient safety and patient care. Therefore, no matter the level of risk involved in improvement science studies, there is a direct impact to patient safety and any delay in approving improvement science studies because of variance in IRB review directly impacts patient care.

In summary, steps and strategies implemented by the ISRN are believed to have helped each of the 14 study sites in successfully achieving timely IRB approvals. Using template IRB documents provided by the Network, each site was given responses to each question on the IRB application reducing the turnaround time for submission. Furthermore, each PIK sent to the site PI included a sample consent form and HIPAA document which eliminated the need for the site PI to develop consent forms of their own, greatly reducing the burden on the site PIs. By providing these templates and documents through the study PIK, each site PI simply needed to transcribe the provided information into their site specific IRB application forms. As indicated earlier, each of the documents in the PIK were reviewed by the UTHSCSA IRB. It can be concluded that providing these previously approved documents as templates, site-specific IRB reviews were able to be completed in a shorter amount of time, resulting in quicker study startup.

## 5. Conclusion

Improvement initiatives have increased dramatically since the IOM report, “To err is human: building a safer health system,” was published [20]. Implementation of improvement research is continuously evolving with new methodologies, new topics of study, and expansion from single site to multisite research. This progress will raise the scientific rigor of these improvement initiatives and will facilitate spread and uptake of effective improvement strategies. Furthermore, with this evolution, challenges have arisen for regulatory agencies, IRBs, and researchers to keep up to date on the interpretation of the regulatory guidances as part of the improvement research initiative. In this report of the ISRN's STAR-2 study, IRB review was conducted in a seemingly streamlined and timely way. However, multiple publications have documented variations of IRB review for research networks, similar to the ISRN, engaged in multisite improvement projects [4, 11–13]. Solutions must be found to facilitate timely and accurate approvals as not to delay the innovation that comes out of quality improvement research. Continued dialogue between improvement researchers and review board chairs is needed for this to happen. By working with a national organization such as the ISRN and the resources it provides, variations in IRB approvals can be limited as evident by the results of this study. However, continued investigation on methods to streamline the implementation of improvement research is warranted. 

## Figures and Tables

**Table 1:** IRB Review type and requirement for documented consent.

Wave	Hospital	IRB type	Review type	Documented consent
1	A	Academic	Expedited	No
1	B	Academic	Expedited	Yes
2	C	Hospital	Expedited	Yes
2	D	Academic	Expedited	Yes
2	E	Hospital	Full Board	Yes
2	F	Hospital	Expedited	Yes
2	G	Hospital	Expedited	No
2	H	Hospital	Expedited	Yes
3	I	Hospital	Expedited	Yes
3	J	Hospital	Full Board	Yes
3	L	Hospital	Expedited	Yes
3	K	Hospital	Expedited	Yes
3	M	Academic	Expedited	Yes
3	N	Hospital	Expedited	Yes

**Table 2:** Study start-up times (in business days).

	Days to submission	Days to approval	Days to study start
All hospitals	45.1 ± 31.8	14 ± 5.7	29.9 ± 10.1
Academic IRB	24.0 ± 22.0	16.3 ± 10.6	22.75 ± 10.2
Hospital-based IRB	54.1 ± 32.2	13.7 ± 2.8	32.7 ± 9.1
